# Pediatric Supracondylar Process Fracture: Radiographic Distinction, Misdiagnosis, and Conservative Management

**DOI:** 10.7759/cureus.68547

**Published:** 2024-09-03

**Authors:** Shrey Nihalani, Katie Frith, Michael Conklin

**Affiliations:** 1 Orthopedic Surgery, University of Alabama at Birmingham, Birmingham, USA

**Keywords:** fracture, variant, supracondylar process, pediatric, diagnosis

## Abstract

A supracondylar process is an embryonic vestigial remnant of the supracondylar foramen. It was intially described as a bone spur of the distal anteromedial humerus. An 11-year-old patient with a history of an asymptomatic mass was initially misdiagnosed with osteochondroma during a visit when five years old. Radiographic analysis revealed a tapered bony prominence with lucency at the base, representing a supracondylar process fracture after a fall. He was treated with a long-arm cast with uneventful healing and symptom resolution.

This case emphasizes the importance of understanding the radiographic differences between the supracondylar process and osteochondroma to prevent misdiagnosis and unnecessary evaluations. These findings suggest that conservative management without neurovascular compromise is acceptable in selected cases.

## Introduction

The supracondylar process is a rare anatomic variant of the humerus affecting up to 0.1-2.7% of individuals [1]. It represents an embryologic vestigial remnant of the supracondylar foramen, which has also been described in certain monkey species, reptiles, climbing animals, and marsupials [2]. The tendinous portion at the distal supracondylar process is believed to be derived from the latissmocondyloideus, a remnant from climbing animals such as primates [2]. It was initially described as a bone spur at the distal anteromedial humerus and is associated with the ligament of Struthers [2-4]. Although it is a rare variant and symptomatic presentation is uncommon, several sequelae have been associated with the supracondylar process, including symptoms of paresthesia and weakness with median and ulnar nerve compression, as well as pain with pronation. This is attributed to its close proximity to the neurovascular structures of the elbow and elevated origin of the pronator teres.

We present the case of an 11-year-old patient with supracondylar process fracture after a bicycle accident. Interestingly, this patient was seen at our institution at five years of age for an asymptomatic mass in that area that was initially misdiagnosed as an osteochondroma. This study aimed to raise awareness of this anatomic variant, discuss how to differentiate it from other entities and delineate treatment options for supracondylar process fractures.

## Case presentation

**Figure 1:**
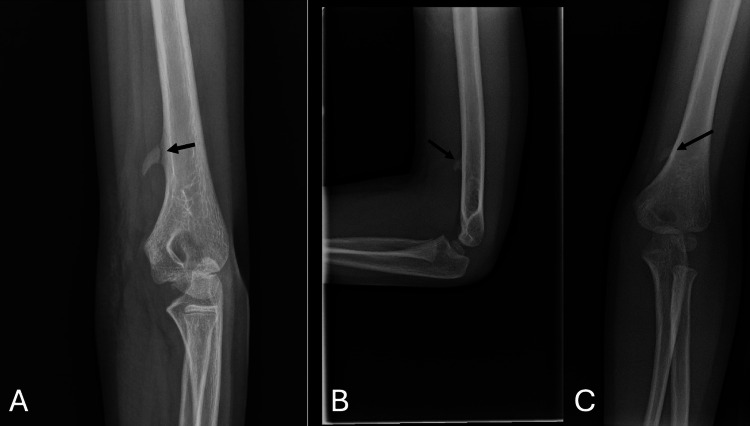
(A) Internal rotation oblique radiograph of left distal humerus in an 11-year-old male shows a supracondylar process with a fracture at its base. Note that the medial humeral cortex is continuous (arrow) so that the medullary canal of the supracondylar process does not continue into the medullary canal of the humerus. (B & C) Anteroposterior and lateral radiographs of the same patient at five years of age (arrows).

An 11-year-old male presented to the orthopedic clinic after a bicycle accident with pain in the distal medial left humerus and superficial abrasions over the medial brachium. He reported tenderness on palpation, with intact neurovascular function. Radiographs revealed a tapered bony prominence extending from the anteromedial distal humerus angled distally and with lucency at its base (Figure 1A). It was noted that films from six years ago were available in the picture archiving and communication system and disclosed a similar lesion without lucency (Figure 1B, 1C). A review of the medical records revealed that he had been seen in our practice by a nurse practitioner for an asymptomatic mass. At that time, it was felt to be an osteochondroma, and a follow-up visit with radiographs was performed months later for reassurance of its benign status.

**Figure 2:**
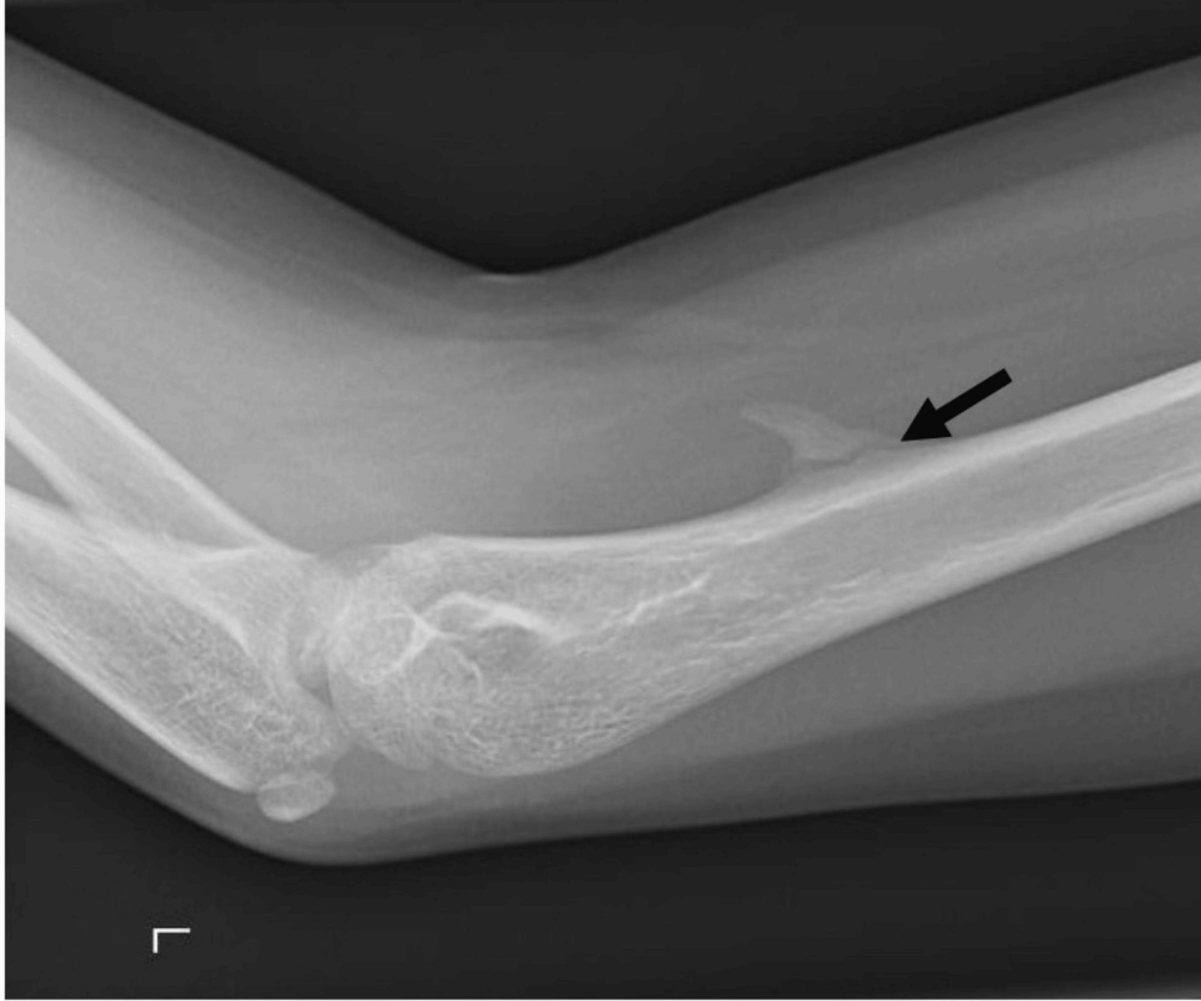
Oblique radiograph four weeks post-injury discloses fracture callus at the base of the lesion (arrow) consistent with healing fracture.

Subsequently, the patient was placed in a long-arm cast. At the month follow-up, there was no tenderness on palpation and full range of motion. Neurovascular function remained intact. Internal oblique radiographs revealed fracture callus formation and no displacement (Figure 2). He was transitioned to a posterior splint for two weeks, with instructions for follow-up as needed. The patient remains asymptomatic.

## Discussion

Our patient was an 11-year-old boy who presented with a fracture through the base of the supracondylar process. This was of interest for several reasons. First, he had been seen in our practice in the remote past, and the lesion was misdiagnosed as an osteochondroma. Osteochondroma is a benign cartilage-capped bone tumor. Solitary lesions are estimated to occur in approximately 3% of individuals [5]. When present on a long bone, they emanate from the metaphysis and point away from the adjacent joint. This helps to distinguish it from the supracondylar process, which points towards the elbow joint. Furthermore, while the medullary canal of an osteochondroma is continuous with the medullary canal of the bone, this is not the case with the supracondylar process (Figure 2) [6]. 

In 1818, Tiedemann described the supracondylar process, a bony hook-shaped structure protruding from the anteromedial humerus and aiming distally [3]. Struthers delineated a ligament extending from the supracondylar process to the medial epicondyle. It has been reported to lead to the median nerve or brachial artery compression, resulting in paresthesias or claudicant arm pain, respectively [4]. Another differential diagnosis is post-traumatic myositis ossificans, but given the history of the patient, this is also unlikely. Understanding these distinguishing features can help to avoid over-evaluation and allay parental concerns since there is a small percentage of osteochondromas that undergo malignant degeneration in adulthood [7]. Plain radiographs are typically adequate to diagnose a supracondylar process. However, ultrasonography and magnetic resonance imaging have been suggested as potential alternatives if radiographs are inconclusive [8].

Another interesting feature of our case is that the patient suffered a fracture of the supracondylar process that healed uneventfully with nonoperative treatment. In 2019, Gamble and Krygier described the case of a five-year-old boy with a supracondylar process fracture who healed with a long arm cast [9]. At the time of their publication, there were six other cases of supracondylar process fractures in children and adolescents, all of which had been treated with surgical excision [9,10]. In our case, the correct diagnosis was made at the time of fracture, and non-operative treatment was adopted based on their recommendations, which resulted in uneventful healing. Based on our experience and that of Gamble and Krygier, in the absence of neurovascular compromise, non-operative treatment with a long-arm cast should be attempted before resorting to surgical treatment [9]. 

## Conclusions

Understanding the radiographic distinction between a supracondylar process and osteochondroma can avoid incorrect diagnosis and over-evaluation. The current case represents an addition to the sparse literature, with only one previously reported case of pediatric supracondylar process fracture treated conservatively. This resulted in uneventful healing. In cases without neurovascular compromise, long-arm cast immobilization should be attempted prior to surgical treatment.
